# Damage Mechanism Analysis of High Field Stress on Cascode GaN HEMT Power Devices

**DOI:** 10.3390/mi16070729

**Published:** 2025-06-22

**Authors:** Shuo Su, Yanrong Cao, Weiwei Zhang, Xinxiang Zhang, Chuan Chen, Linshan Wu, Zhixian Zhang, Miaofen Li, Ling Lv, Xuefeng Zheng, Wenchao Tian, Xiaohua Ma, Yue Hao

**Affiliations:** 1 School of Electronics & Mechanical Engineering, Xidian University, Xi’an 710071, China; 23041212658@stu.xidian.edu.cn (S.S.); 23041212555@stu.xidian.edu.cn (W.Z.); 22041212821@stu.xidian.edu.cn (X.Z.); 22041212707@stu.xidian.edu.cn (C.C.); 22043222985@stu.xidian.edu.cn (L.W.); 24041212505@stu.xidian.edu.cn (Z.Z.); 24041212453@stu.xidian.edu.cn (M.L.); wctian@xidian.edu.cn (W.T.); 2 State Key Discipline Laboratory of Wide Bandgap Semiconductor Technology, School of Microelectronics, Xidian University, Xi’an 710071, China; llv@xidian.edu.cn (L.L.); xfzheng@mail.xidian.edu.cn (X.Z.); xhma@xidian.edu.cn (X.M.); yhao@xidian.edu.cn (Y.H.)

**Keywords:** Cascode GaN HEMT power device, off-state high field stress, noise

## Abstract

A series of problems, such as material damage and charge trap, can be caused when GaN HEMT power devices are subjected to high field stress in the off-state. The reliability of GaN HEMT power devices affects the safe operation of the entire power electronic system and seriously threatens the stability of the equipment. Therefore, it is particularly important to study the damage mechanism of GaN HEMT power devices under high field conditions. This work studies the degradation of Cascode GaN HEMT power devices under off-state high-field stress and analyzes the related damage mechanism. It is found that the high field stress in the off-state will generate a positive charge trap in the oxide layer of the MOS device in the cascade structure. Moreover, defects occur in the barrier layer and buffer layer of GaN HEMT devices, and the threshold voltage of Cascode GaN HEMT power devices is negatively shifted, and the transconductance is reduced. This study provides an important theoretical basis for the reliability of GaN HEMT power devices in complex and harsh environments.

## 1. Introduction

GaN HEMT devices have been widely used in the fields of defense electronic communication and military radar because of their excellent characteristics, such as high electron mobility, high voltage, and high-power resistance [1,2,3,4]. However, semiconductor devices in these areas often need to operate in the high-pressure environment for a long time [5,6,7,8]. In the application of high-voltage switches, the high field stress of GaN HEMT power devices in the off-state will cause a series of reliability problems, such as material damage, charge traps, and so on. These problems will not only affect the long-term stability of the device but also directly affect the safe operation and life of the entire power electronic system and thus seriously threaten the stability of the equipment [9,10,11,12].

In high-voltage and high-frequency power electronics systems, Cascode GaN HEMT power devices which are made by Transphorm company (Goleta, CA, USA) have become one of the mainstream choices in the industry due to their compatibility with traditional silicon-based drives, high voltage resistance, and ease of use. As the system voltage increases to 650 V or even 1200 V, the reliability problem caused by high field stress in the off-state becomes increasingly prominent. The damage mechanism of GaN and MOS devices under high field stress has been studied extensively. Joh J. found that GaN HEMT devices which are made by Microsystems Technology Laboratories (Cambridge, MA, USA) in MIT have a critical voltage. When the applied stress exceeds this voltage, crystal damage occurs in the device material, resulting in permanent degradation of performance [13]. To reveal the whole process of device performance degradation, the researchers studied the changes inside the device under different stress conditions and explained the causes of crystal damage. By studying the degradation behavior of GaN HEMT devices under high field stress, Wang Y.S. found that high field stress would cause structural damage near the gate of the device, and many defects would accumulate in the barrier layer before the damage occurred [14]. Yao Z.W. found through transient current tests that long-term high field stress would significantly increase the intrinsic defect concentration in the barrier layer and would not change the defect energy level [15]. Onishi found that degradation of NMOS devices which are made by IBM Microelectronics (Armonk, NY, USA) under gate stress is mainly caused by charge capture in the HfO_2_ layer [16]. A. Benabdelmoumene tested the negative gate pressure stress of NMOS devices and found that two trap charges with opposite states were formed in the oxide layer [17]. Zhang X.X. conducted a negative gate compressive stress test on the Cascode GaN HEMT device and found that the pre-applied stress slightly increased the transconductance of the Cascode GaN HEMT device [18]. Cai X.Z. et al. analyzed the variation of internal defects in cascode GaN devices after irradiation through low-frequency noise testing and found that the defect density inside the devices decreased after total ionizing dose irradiation [19]. Lv L. conducted an analysis of GaN HEMT devices after heavy-ion incidence using low-frequency noise testing and found that heavy-ion radiation led to an increase in the internal defect density of the devices, causing severe damage to the devices [20].

Although scholars at home and abroad have extensively studied the stress damage mechanism of MOS devices and GaN HEMT devices, these studies mainly focus on the degradation of a single device, and there is less mechanism explanation for the degradation mechanism of Cascode GaN HEMT power devices composed of the two cascades under high field stress. The high-voltage degradation of cascode seriously affects its reliability in high-voltage environments. However, the degradation mechanism analysis of cascode in high-voltage environments is less. Thus, we have a deep study on the threshold voltage and transconductance degradation mechanisms under off-state high-field stress. Moreover, we have a deep analysis of the physical explanation of threshold voltage and transconductance degradation mechanisms. This study can help with the degradation analysis of cascode in high-voltage environments and provide a theoretical basis for the high-voltage reliability design of Cascode GaN HEMT power devices.

## 2. Experiments and Devices

To study the degradation mechanism of GaN HEMT devices under off-state high field stress, off-state high-field-stress experiments were conducted on GaN HEMT devices. In the off-state high-field-stress experiment, 300 V, 400 V, and 500 V voltages are applied to the drains of Cascode GaN HEMT power devices, respectively, and the voltage duration is 10,000 s. During the experiment, the gate voltage was set to 0 V so that the devices remain in the off-state. The electrical characteristics of the device were tested at 200 s, 500 s, 1000 s, 2000 s, 5000 s, and 10,000 s when the voltage was applied. The output and transfer characteristics of the device were measured using the 1505 Keysight semiconductor parameter tester, and changes in the device threshold voltage, transconductance, and saturation leakage current were extracted. The mechanism analysis was based on the device structure and simulation. A low-frequency noise test was combined to verify the mechanism analysis.

**Figure 1:**
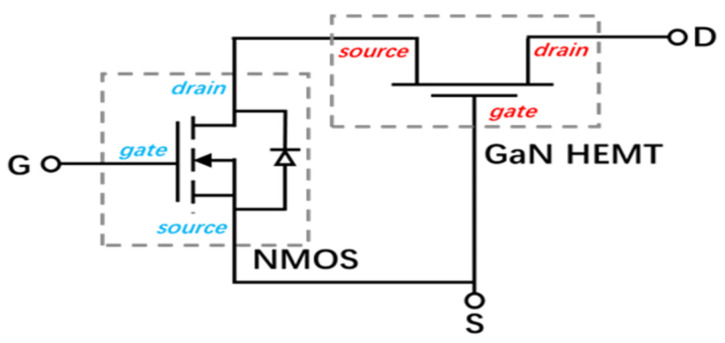
Cascode GaN HEMT power device structure.

Figure 1 shows the structure of the Cascode GaN HEMT device, which is composed of an enhanced MOSFET device and a depletion GaN HEMT device. The drain of the MOSFET device is connected to the source of the GaN HEMT device, and the source of the Cascode GaN HEMT power device is composed of the source of the MOS device and the gate of the GaN HEMT device. The gate of the Cascode GaN HEMT power device is composed of the gate of the MOS device. The drain of the Cascode GaN HEMT power device is composed of the drain of the GaN HEMT device.

**Figure 2:**
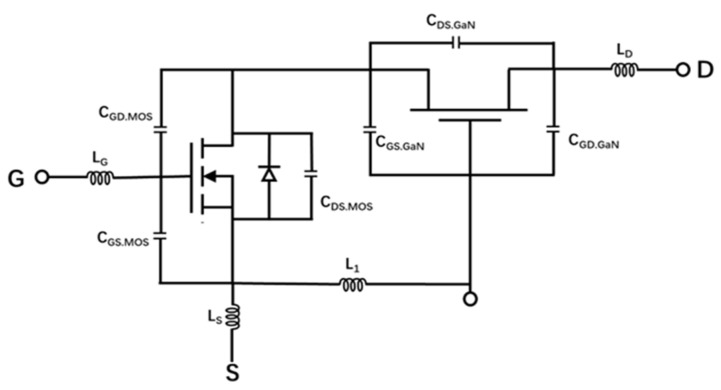
The equivalent capacitance and circuit of Cascode GaN HEMT.

Figure 2 shows the equivalent capacitance and circuit of Cascode GaN HEMT. During the turn-on process of the cascode circuit, C_DS.MOS_ and C_GS.GaN_ start to decrease. Moreover, V_GS.GaN_ is less negative. As time increases, V_GS.GaN_ is larger than V_TH.GaN_, and the entire circuit turns on. During the turn-off process of the cascode circuit, V_GS.MOS_ is less than V_TH.MOS_, and the MOS device is in the off-state. The voltage V_S.GaN_ increases, which is the same as V_D.MOS_. As time goes on, V_GS.GaN_ becomes less than V_TH.GaN_, and the GaN device is turned off. The voltage division between the MOS device and the GaN device is approximately equal to the voltage division between C_DS.MOS_ and C_DS.GaN_. C_DS.MOS_ and C_DS.GaN_ are in series relative to the entire circuit, and C_DS.GaN_ bears most of the voltage.

**Figure 3:**
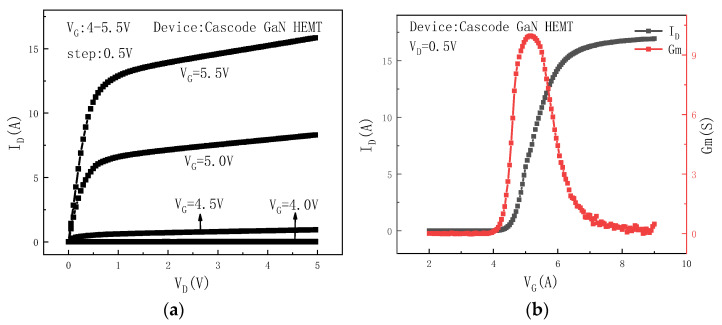
(**a**) The output curve of the Cascode GaN HEMT power device. (**b**) The transfer curve and transconductance curve of the Cascode GaN HEMT power device.

In this paper, the off-state high-field stress is applied to the drain of the Cascode GaN HEMT power devices. When high voltage is applied to the drain, the gate voltage is less than the threshold voltage of the device. Hence, the MOS device is in the off-state, and a voltage drop is formed between the drain and the source of the MOS device. The V_DS.MOS_ is positive. The drain electrode of the MOS device is directly connected to the source electrode of the GaN device, and the source electrode of the MOS device is directly connected to the gate electrode of the GaN device. Thus, V_GS.GaN_ is equal to V_SD.MOS_, which is negative. V_GS.GaN_ is more negative than the threshold voltage of the GaN HEMT device, so the GaN HEMT device is also in the off-state. When the gate voltage is higher than the threshold voltage of the device, the MOS device is on. Both the C_DS.MOS_ and C_GS.GaN_ begin to decrease. The V_GS.GaN_ increases and gradually exceeds the threshold voltage of the GaN HEMT device, causing the GaN HEMT device to conduct as well. The rated breakdown voltage of the device is 650 V, and the threshold voltage is 4.43 V. Figure 3 shows the basic characteristics of the Cascode GaN HEMT power device.

## 3. Analysis of Experimental Results

### 3.1. Influence of Different Drain Voltages on Electrical Characteristics of Devices

To explore the degradation of Cascode GaN HEMT power devices under off-state high-field stress, 300 V, 400 V, and 500 V high-field stress are applied at the drain electrode. Figure 3 shows the degradation and recovery of threshold voltage over time under different off-state high-field stress. The gate of the Cascode GaN HEMT power device is the gate of the enhanced MOS device, and the cascaded GaN HEMT device is the depletion type; thus, the enhanced MOS devices play a role in controlling the switching of the entire circuit. Hence, the threshold voltage of the Cascode GaN HEMT power device is the threshold voltage of the enhanced MOS device. In the off-state stress test, the MOS device and GaN HEMT device are in the off-state, and the entire circuit can be approximately regarded as two capacitors connected in series together, and the drain voltage is shared by the MOS device and GaN HEMT device. Because the channel resistance of GaN devices is much larger than the channel resistance of MOS devices, GaN HEMT devices receive much of the voltage, and MOS devices receive the minority of the voltage. The gate voltage division of MOS devices is relatively small, so the devices will not break down [21]. In a series capacitor circuit, with the rise of the total voltage, the voltage division of each capacitor also goes up. Therefore, as the drain voltage of the Cascode GaN power device rises, the voltage division of the MOS device grows.

**Figure 4:**
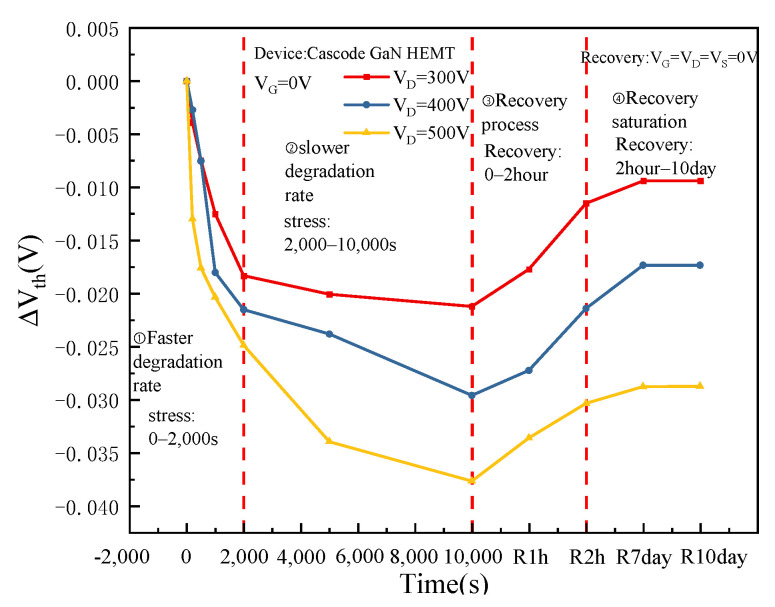
The threshold voltage changes the curve of devices with time.

The Cascode GaN HEMT power device controls the opening of the depletion GaN HEMT device through the enhanced MOS device. Therefore, when studying the threshold voltage degradation of Cascode GaN power devices, the research mainly focuses on MOS devices in the cascaded structure. It can be seen from Figure 4 that the threshold voltage degradation rate is fast in the initial period of stress application, and the threshold voltage degradation rate slows down after a period. The degradation of threshold voltage increases with the increase in drain voltage. At the initial stage of stress removal, the threshold voltage recovery rate is fast. After a period, the threshold voltage recovery reaches saturation and is lower than the initial level, indicating that permanent damage has occurred in the devices.

**Figure 5:**
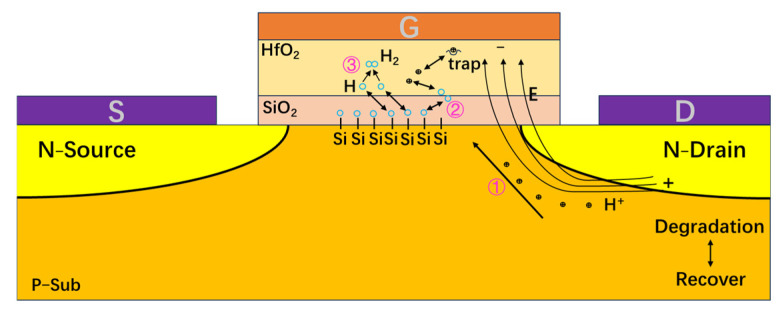
Mechanism diagram of threshold voltage degradation of MOS devices.

In the off-state high-field-stress experiment, the drain voltage of the MOS device is higher than the gate voltage of the MOS device, and there is a negative bias electric field under the gate of the MOS device. As shown in ① in Figure 5, the holes in the substrate move towards the gate under the action of the electric field, which has an impact on the Si-H bonds at the interface between the oxide layer and the substrate [22]. With the increase in time, part of the Si-H bonds is destroyed. Si- and H atoms are produced as shown in ② in Figure 5. Si- atoms form interface traps, and H atoms will spread to the gate direction. Part of the H atoms will lose electrons to form hydrogen ions, and hydrogen ions will shift to the gate direction under the action of the negative bias electric field. When passing through the oxide layer, part of the hydrogen ions is captured by the oxide layer traps to form positive charge traps, which leads to the degradation of threshold voltage.

**Figure 6:**
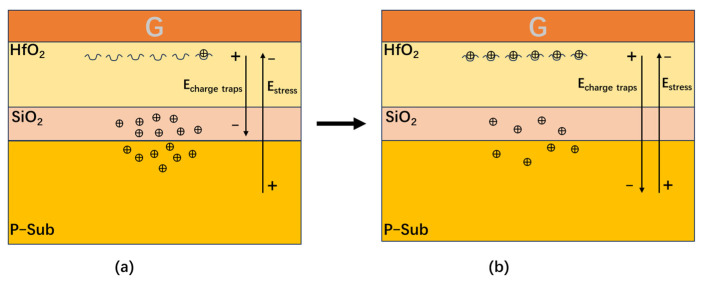
A schematic diagram of the internal changes in the MOS device over time. (**a**) the rapid degradation stage of the threshold voltage. (**b**) the saturation stage of threshold voltage degradation.

The positive charge traps formed in the oxide layer will prevent hydrogen ions from shifting towards the gate. At the initial stage of stress application, the positive charge traps formed in the oxide layer are fewer, and the obstacles to hydrogen ions shifting toward the gate are weak. Figure 6a shows the electric field under the gate during the rapid degradation stage of the threshold voltage when stress is first applied. At this time, the electric field generated by the positive charge traps is smaller than that generated by the stress. The hydrogen ions shift toward the gate direction more easily, and the oxide trap is easy to trap hydrogen ions to form positive charge traps, and the degradation rate of the threshold voltage of the device is faster. With the increase in time, more and more positive charge traps are formed in the oxide layer, and the obstacle of hydrogen ions shifting toward the gate becomes stronger and stronger, and hydrogen ions shifting toward the gate becomes difficult. Figure 6b shows the electric field under the gate during the saturation stage of threshold voltage degradation after stress is applied for a period. At this time, the electric field generated by the positive charge traps is equal to that generated by stress. The oxide traps are not easy to capture hydrogen ions to form positive charge traps, and the degradation rate of the threshold voltage of the device slows down.

After the stress is removed, the recovery is mainly caused by the detrapping of some hydrogen ions. Some H atoms return to the interface between the oxide layer and the substrate to form Si-H bonds with the Si suspension bonds, and the device threshold voltage begins to recover. Because a small number of H atoms will combine with each other to form H^2^ during the process of diffusing towards the gate, as shown in ③ in Figure 5. Therefore, the threshold voltage of the devices still does not reach the initial level when they recover to the saturation state. The high-field stress causes permanent damage at the gate oxide layer and the interface between the oxide layer and the substrate of the devices. When the drain voltage of the Cascode GaN HEMT power device rises, the total voltage of the circuit grows. The voltage division at the gate of the MOS device increases, and the degradation of the device’s threshold voltage becomes more pronounced.

To verify the degradation mechanism of threshold voltage of MOS devices in cascade structure under negative gate stress, the method of adding donor defects in the oxide layer of MOS devices was adopted. The donor defects were used to capture holes to form positive charge traps to simulate the trapping of hydrogen ions by the oxide layer traps under the high field stress in the off-state. Because the threshold voltage of the MOS device determines the threshold voltage of the cascode. Hence, the threshold voltage of a MOS device is a very important parameter. The threshold voltage of the MOS simulation model is 4.41 V, which is in good agreement with the actual devices’ threshold voltage of 4.43 V. In the simulation, the oxide layer width-to-length ratio of the MOS device is 1000 nm/100 nm. Relevant physical models are added in the simulation to more realistically mimic practical scenarios. Under the action of a high electric field, carriers collide with lattice atoms to cause impact ionization, generating high-density charges. Therefore, an impact self-model and a Fermi–Dirac carrier number statistics model, which more accurately calculates high-concentration carriers, are added. Since doping concentration affects mobility, a concentration-dependent mobility model is included. The high electric field saturation influences the charge drift velocity, so a parallel electric field-dependent model is added. Due to the high source-drain doping concentration in the MOSFET device, the bandgap narrowing model is needed to describe the bandgap narrowing effect under high doping concentration.

**Figure 7:**
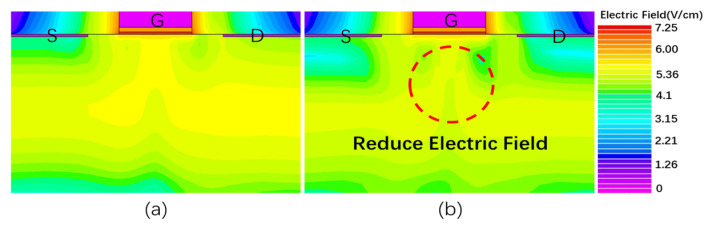
Electric field distribution inside MOS device. (**a**) Conventional device. (**b**) Donor trap device.

Figure 7 shows the comparison of the internal simulation electric field distribution between a conventional MOS device and a MOS device with donor defects. As can be seen from Figure 7, adding donor defects to the oxide layer of the MOS device will reduce the electric field of the substrate below the gate of the MOS device. This is because the trapping of holes by donor defects in the oxide layer decreases the concentration of holes at the interface between the oxide layer and the substrate. This results in a decrease in the internal electric field of the substrate below the gate, which corresponds to ① in Figure 5. The reduction in the electric field of the substrate below the gate will reduce the source-substrate voltage *V_SB_*. Formula (1) is the calculation formula of the threshold voltage of the MOS device. From Equation (1), a decrease in *V_SB_* reduces $\frac{\sqrt{2q\in_{s}N_{A}(2\emptyset_{F}+V_{SB})}}{C_{0x}}$ in Equation (1), resulting in a decrease in *Vth* and a negative shift in the device threshold voltage. It is consistent with the MOS device threshold voltage degradation mechanism presented in Figure 5.

(1) $$V_{th}=V_{FB}+2\emptyset_{F}+\frac{\sqrt{2q\in_{s}N_{A}(2\emptyset_{F}+V_{SB})}}{C_{ox}}$$

where *V_FB_* is the flat band voltage; *Φ_F_* is the Fermi potential; *q* is the electron charge; *ε_S_* is the dielectric constant; *N_A_* is the substrate doping concentration; *V_SB_* is the source-substrate voltage; and *C_OX_* is the oxide layer capacitance.

**Figure 8:**
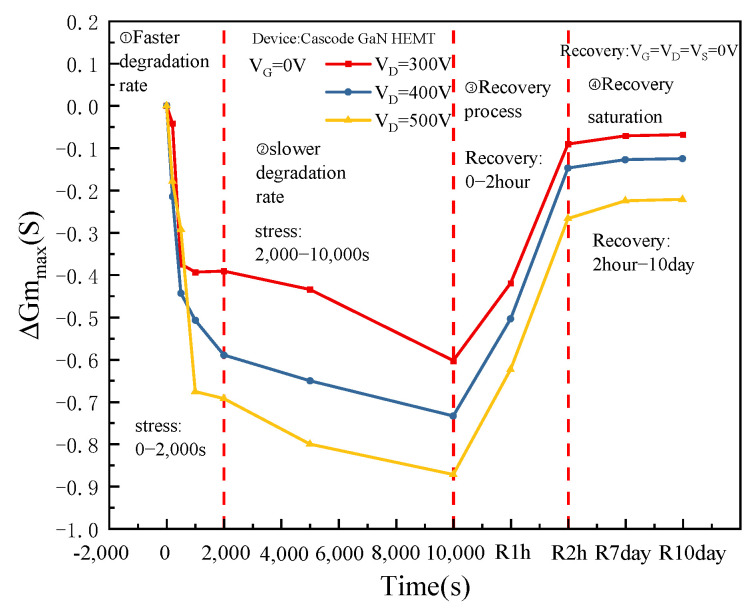
The maximum transconductance curve of the devices with time.

When constant off-state high field stress is applied to the drain electrode, the device transconductance degrades. Figure 8 shows the transconductance curve of the device varies with time. It can be seen from Figure 8 that the transconductance degradation rate is fast in the initial period of stress application and slows down after a period. Moreover, the degree of transconductance degradation increases with the increase in drain voltage. At the beginning of stress removal, the transconductance recovery rate is fast. After a period, the transconductance recovery reaches saturation, but it is lower than the initial level, which also proves that permanent damage occurs inside the device.

**Figure 9:**
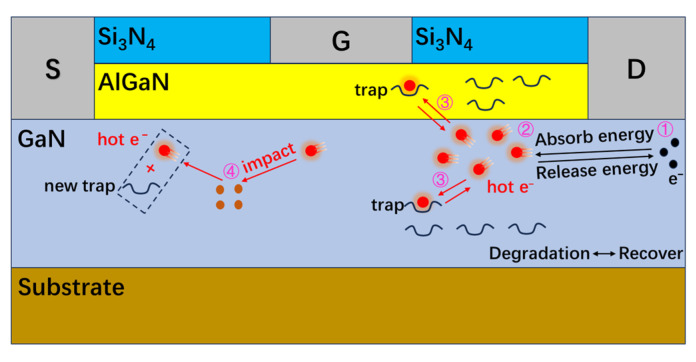
Transconductance degradation mechanism of GaN HEMT device.

The transconductance of the Cascode GaN HEMT power device is obtained by comparing the drain current of the GaN HEMT device to the gate voltage of the MOS device. The threshold voltage of MOS devices shifts negatively. When the gate voltage is the same, the device’s turn-on degree is higher and the output current is larger. However, the threshold voltage shift of MOS devices is relatively small. The change in drain current caused by the threshold voltage shift is also relatively small. Therefore, when studying the transconductance degradation of Cascode GaN HEMT power devices, the research mainly focuses on GaN HEMT devices in the cascaded structure. Figure 9 shows the transconductance degradation mechanism of GaN HEMT devices under off-state high-field stress.

In the off-state stress test, the MOS device and GaN HEMT device are both in the off-state. The entire circuit can be approximately two capacitors in the series together, and the drain voltage is shared by the MOS device and GaN HEMT device. Because the channel resistance of the GaN HEMT device is much larger than the channel resistance of the MOS device, the high field stress is mainly shared by the GaN HEMT device. As shown in Figure 9 ①, a long time of high field stress will lead to a small number of leaking electrons under the drain electrode [23]. When these electrons are near the drain, some electrons obtain enough energy to become hot electrons, as shown in Figure 9 ②. Among them, the hot electrons with higher energy escape after being subjected to lattice elastic collisions, as shown in Figure 9 ③. At the same time, high field stress will activate traps in the barrier layer and buffer layer of the GaN HEMT device. These traps capture the leaked electrons, causing the output current to decrease and the transconductance of the Cascode GaN HEMT power device to be reduced.

The degradation of the threshold voltage reduces the gate voltage required for MOS devices to turn on, but the change in the threshold voltage is relatively small. The change in the threshold voltage leads to a small change in the current output of the device and has a small impact on transconductance. Therefore, the decrease in transconductance is mainly caused by internal defects in GaN HEMT devices.

**Figure 10:**
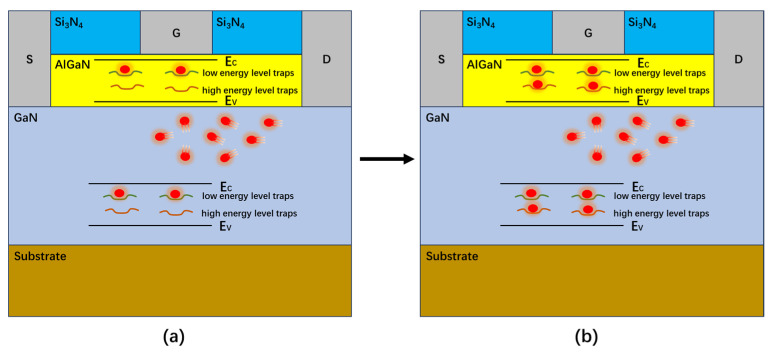
A schematic diagram of the internal changes in the GaN device over time. (**a**) the rapid degradation stage of transconductance. (**b**) the saturation stage of transconductance degradation.

At the initial stage of stress application, the more easily activated low-energy-level traps in the device are activated. Figure 10a shows the electrons are obtained by internal defects of the device during the rapid degradation stage of transconductance. Many electrons are captured by low-energy-level traps, and the transconductance degradation rate is fast. With the increase in time, the low-energy-level traps are all activated and have captured electrons, and the high-energy-level traps begin to be activated. Figure 10b shows that the electrons are obtained by internal defects of the device during the saturation stage of transconductance degradation. The electrons begin to be captured by the high-energy-level traps, and the transconductance degradation rate slows down.

After the stress is removed, the low-energy-level traps, which are easier to deactivate, start to deactivate. The low-energy-level traps deactivate faster, and the transconductance recovery rate is faster. After a period, all low-energy-level traps are inactivated, while high-energy-level traps begin to be inactivated. The high-energy-level traps are slowly inactivated, and the transconductance recovery rate slows down. Because hot electrons will generate permanent new traps inside the device after escaping from the lattice collision, as shown by ④ in Figure 9. The transconductance of the device still does not reach the initial level when recovering saturation. The high field stress causes permanent damage inside the device.

To verify the transconductance degradation mechanism of the GaN device in the cascade structure under the off-state and high field, the acceptor defects were added to the barrier layer and buffer layer to simulate the barrier layer traps and buffer layer traps trapping electrons. Because the drain current of the GaN device determines the drain current of the cascode. Hence, the drain current of a GaN device is a very important parameter. When the drain voltage and gate voltage are 5 V, the drain current of the GaN simulation model is 5.33 A, which is in good agreement with actual devices (5.35 A) under the same conditions. In the simulation, the gate length of the GaN device is 2 μm, the AlGaN barrier layer thickness is 15 nm, and the buffer layer thickness is 1.95 μm. To simulate practical conditions more realistically, appropriate physical models need to be selected in the simulation. The polarization model can simulate the polarization effect on GaN devices, using psp.scale, polar.scale, and piezo.scale to represent polarization charges caused by spontaneous polarization, total polarization, and piezoelectric polarization. Under the action of a high electric field, carriers collide with lattice atoms to generate more electron–hole pairs, and the impact self-model can well simulate this process. The Shockley–Read–Hall recombination model can effectively simulate the recombination process of partial electron–hole pairs with defects inside the device. Moreover, the Fermi–Dirac carrier number statistics model is suitable for scenarios requiring number statistics of many particles. Since carriers will generate many electron–hole pairs via impact ionization under high electric fields, introducing this model can more accurately count the number of carriers after ionization.

**Figure 11:**
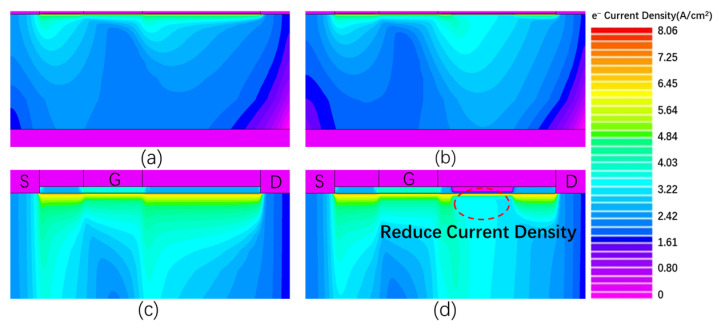
Comparison of electron concentrations in GaN HEMT devices. (**a**) Conventional device; (**b**) defective device; (**c**) partial amplification of conventional device; and (**d**) partial amplification of defective device.

Figure 11 shows the comparison of electron concentration in the conventional GaN HEMT device and the device with defects. It can be seen from Figure 11 that the addition of acceptor defects in barrier and buffer layers of the GaN HEMT device will reduce the concentration of channel electrons. Because electrons in the channels are obtained by the traps in the barrier layer and the buffer layer, which corresponds to ③ in Figure 9. A decrease in channel electron concentration will reduce the output current and transconductance of the device. This is consistent with the transconductance degradation mechanism of the GaN HEMT device in Figure 9.

### 3.2. Influence of Different Drain Voltages on Noise of Device

**Figure 12:**
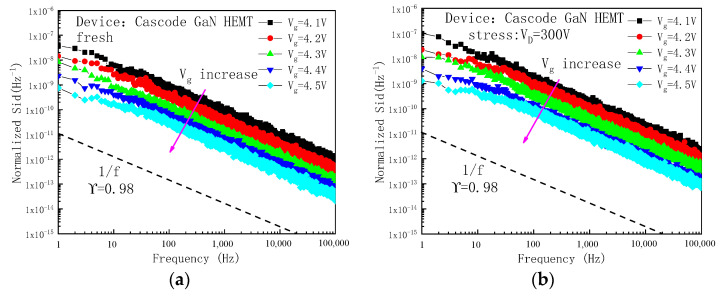
Normalized low frequency noise curve of the devices. (**a**) Fresh device. (**b**) Device with a stress of 300 V applied to the drain.

The noise test of Cascode GaN HEMT power devices before and after stress was carried out by the FS-Pro series semiconductor parameter test system, and the defects introduced by high field stress in the devices were indicated. In Cascode GaN HEMT power devices, the gate oxide layer traps of the MOS device and the traps in the barrier layer and the buffer layer of the GaN HEMT device will obtain electrons, causing changes in the number of electrons in the channel, which will cause the number of electrons in the channel to fluctuate. When a small voltage is applied to the drain, the change in the number of electrons will be shown by the change in drain current. In the noise test, constant small drain voltage is applied to the drain electrode, and the gate voltage gradually increases from around the threshold voltage. Because the gate voltage is near the threshold voltage and the device is in a half-open state, a small current will be generated inside the device, and the internal current of the device will change with the change in the gate voltage [24]. The drain current, which included the noise current, can be processed to achieve a relationship between power spectral density (PSD) and frequency. Figure 12 shows the normalized low-frequency noise curve of the device. It can be found from Figure 12 that as the gate voltage increases, the noise gradually decreases. The value of the frequency index factor γ obtained through data fitting is 0.98, which is very close to 1, belonging to the characteristics of *1/f* noise. Moreover, 1/f noise is caused by the change in the electron number within the channel. Defects in both the MOS and GaN devices trap electrons and can change the electrons’ number. The defects in the MOS device have an influence on the threshold voltage. The threshold voltage change will have an influence on the drain current. Moreover, the defects in the GaN device will trap the electrons in the channel, which also has an influence on the drain current.

According to the classical tunneling theory, the relationship between drain current Sid and flat-band voltage noise power spectral density *S_vfb_* is as follows:

(2) $$\frac{Sid}{I_{ds}^{2}}={\left(\frac{g_{m}}{I_{ds}}\right)}^{2}\cdot S_{vfb}$$

where *g_m_* and *I_ds_* are, respectively, device transconductance and drain current, which are obtained by the test system. The relationship between defect density *N_t_* and flat band voltage noise power spectral density *S_vfb_* is as follows:

(3) $$N_{t}=\frac{WLC_{ox}^{2}f}{q^{2}kT\lambda }S_{vfb}$$

where *W*, *L*$,and\;\;C_{ox}^{2}$ correspond to the width, length, and barrier layer capacitance of the device, respectively. *f* is the test frequency. *q* is the charge of the electron. *k* is the Boltzmann constant. *T* is the Kelvin temperature. *λ = 0.5* nm is the tunneling factor. *S_vfb_* is flat band voltage noise power spectral density. *N_t_* is the defect density of the device.

It can be seen from Formula (3) that for the same device, the flat band voltage noise power spectral density *S_vfb_* is proportional to the defect density *N_t_* of the device when the environment temperature and test frequency are unchanged.

**Figure 13:**
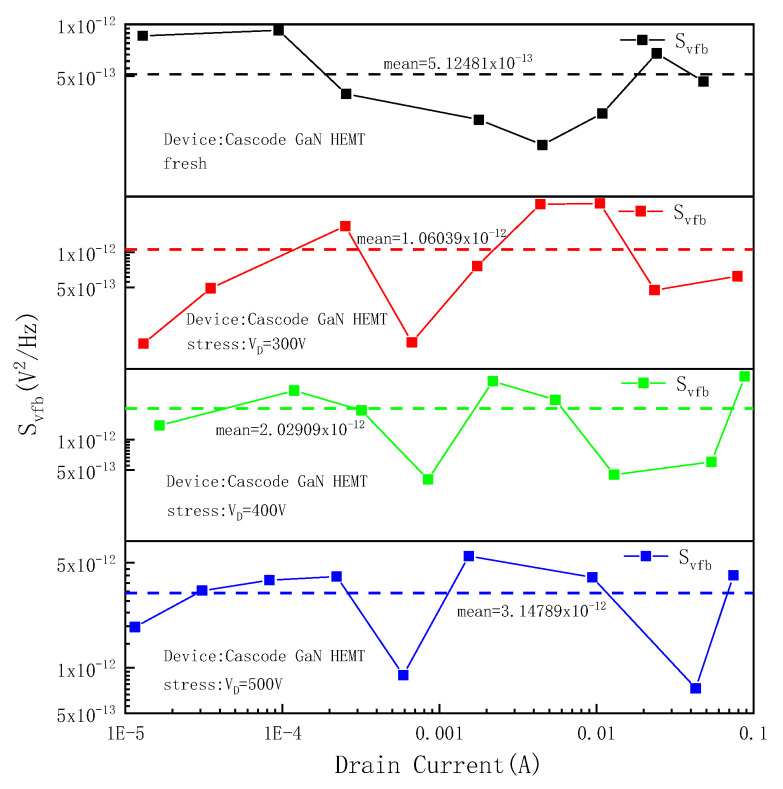
Flat band voltage noise power spectral density *S_vfb_* and the average value of devices.

To study the influence of off-state high field stress on the internal defect density of the device, the normalized drain current and transconductance of the same frequency in the low-frequency noise test of the Cascode GaN HEMT power device were extracted. The flat band voltage noise power spectral density *S_vfb_* of the device with different drain currents was calculated, and its average value was taken to characterize the internal defect density of the device. Figure 13 shows the flat band voltage noise power spectral density *S_vfb_* and its average value of the device before and after stress application at a frequency of 10 Hz. It can be seen from Figure 13 that applying high field stress to the drain will increase the flat band voltage noise power spectral density *S_vfb_* of the device, and the defect density inside the device will increase. Moreover, the higher the drain voltage is, the higher the defect density inside the device will be. The analysis results of low-frequency noise are consistent with the mechanism analysis mentioned above.

## 4. Conclusions

In this paper, the damage mechanism of a Cascode GaN HEMT power device under off-state high-field stress is studied by combining experiment with simulation, and the defects inside the device are indicated by a low-frequency noise test. It is found that when the Cascode GaN HEMT power device is in the off-state, long-term drain high-field stress will degrade the electrical characteristics of the device. The gate of the Cascode GaN HEMT power device is the gate of the MOS device. The oxide layer traps of MOS devices obtain hydrogen ions that drift towards the gate due to the negative bias electric field of the gate, causing the holes in the substrate to drift towards the interface between the oxide layer and the substrate. Therefore, the internal electric field of the substrate below the gate decreases, resulting in a reduction in the source-substrate voltage V_SB_ and a negative drift of the device threshold voltage. The transconductance of the Cascode GaN HEMT power device is determined by the drain current of the GaN HEMT device compared with the gate-source voltage of the MOS device. The drift of the threshold voltage has a small impact on transconductance, so the transconductance of the device is mainly affected by the drain current of the GaN HEMT device. Because the high field stress activated traps in the barrier layer and buffer layer of the GaN HEMT device. These traps obtain escaping electrons, causing the output current to decrease and resulting in a reduction in the transconductance of the device. Finally, the low-frequency noise test shows that the internal noise of the device belongs to 1/f noise. Moreover, the higher the voltage of the drain, the higher the defect density in the device.

## Data Availability

The data presented in this study are available on request from the corresponding author.
